# Effects of Loneliness and Stress Management on Perceived Stress in Older Adults

**DOI:** 10.1093/geroni/igab046.3323

**Published:** 2021-12-17

**Authors:** Angela Gifford, Hayley Fouche, Janelle Beadle

**Affiliations:** 1 University of Wisconsin-Madison, Madison, Wisconsin, United States; 2 University of Nebraska at Omaha, Omaha, Nebraska, United States

## Abstract

In older adulthood, individuals may experience acute and chronic stressors, such as the loss of independence, mobility, or the experience of chronic diseases. Loneliness is also a concern in older adulthood as many experience the loss of close others and smaller social networks. Loneliness is well-established as being associated with higher stress levels in younger adults, but there are mixed findings on the impact in older adulthood. Furthermore, while older adults may engage in behaviors designed to reduce and manage stress, it is not known whether these behaviors modulate the relationship between loneliness and chronic stress. The current study examined the relationship between loneliness and stress in older adulthood and the degree to which stress-management moderated this relationship (note: data collected prior to COVID-19 pandemic). We hypothesized that loneliness and perceived stress would be positively associated, but that stress-management would moderate this relationship. Participants included 40 healthy older adults (Mage= 69.18, Range:55-86yrs; 29 females) who completed the UCLA-Loneliness Scale, the Perceived Stress Scale, the NEO-FFI (to assess neuroticism), and demographic information about participation in stress management activities. We found that loneliness was significantly associated with chronic stress, r(38) = .517, p < .001. Furthermore, loneliness and neuroticism were significant predictors of perceived stress, F(2, 37) = 9.618, p < .001, R2 = .342. These findings demonstrate that loneliness is a significant predictor of perceived stress in older adulthood and point to a need for further exploration of effective stress-management tools in later life.

